# Moxibustion for anorexia in COVID-19: A protocol for systematic review and meta-analysis

**DOI:** 10.1097/MD.0000000000028894

**Published:** 2022-02-25

**Authors:** Xingxin Wang, Yi Hou, Lin Ding, Xiaojun Zheng, Yawen Sheng, Qiaoru Yu, Xiaoyun Bi, Jiguo Yang

**Affiliations:** College of Acupuncture and Massage, Shandong University of Traditional Chinese Medicine, Jinan, People's Republic of China.

**Keywords:** corona virus disease 2019, meta-analysis, moxibustion, protocol, systematic review, traditional Chinese medicine

## Abstract

**Background:**

As the outbreak of coronavirus disease 2019 (COVID-19), caused by severe acute respiratory syndrome coronavirus 2 (SARS-CoV-2), has rapidly spread over the world, the World Health Organization has declared the outbreak of COVID-19 an international public health emergency. Besides typical respiratory symptoms and signs of COVID-19, digestive symptoms and liver injury have been frequently reported during the course of the disease. The purpose of this study was to evaluate the efficacy and safety of moxibustion in the treatment of anorexia in patients with COVID-19.

**Methods:**

According to the retrieval strategies, randomized controlled trials on moxibustion therapies for C19-A will be obtained from the China National Knowledge Infrastructure, WanFang Data, Chinese Scientific Journals Database, PubMed, Embase, and Cochrane Library, regardless of publication date or language. Studies will be screened based on inclusion and exclusion criteria, and the Cochrane risk bias assessment tool will be used to evaluate the quality of the literature. The network meta-analysis will be performed with the Markov chain Monte Carlo method and carried out with Stata 14.2 and WinBUGS 1.4.3 software. Ultimately, the quality of the evidence obtained from the results will be evaluated.

**Results:**

This study will evaluate whether moxibustion therapy can effectively treat anorexia in patients with COVID-19.

**Conclusion:**

This study will provide evidence for whether moxibustion therapy is beneficial to the treatment of anorexia in patients with COVID-19.

**Trial registration number:**

CRD42022302499

## 1 Introduction

The medical havoc wreaked by the novel severe acute respiratory syndrome coronavirus (SARS-CoV-2) has grown exponentially to pandemic proportions within a short span of time, starting in December 2019. Fever, dyspnea, weakness, shivering, C-reactive protein, fatigue, dry cough, anorexia, anosmia, ageusia, dizziness, sweating, and age were the most important symptoms of COVID-19 infection. The prevalence of gastrointestinal symptoms ranged from 6.8% to 61.3%, including diarrhea (8.14%–33.7%), nausea/vomiting (1.53%–26.4%), anorexia (12.1%–40.0%) and abdominal pain (0%–14.5%).^[1]^ In the study of Lin et al, 95 patients (50 women and 45 men) from Zhuhai, China, with COVID-19 and mean age of 45.3 ± 18.3 years, 58 (61.0%) presented with gastrointestinal symptoms, in which 11 (11.6%) at hospital admission and 47 (49.5%) during hospitalization. The latter probably had the condition aggravated by use of drugs, such as antibiotics. The main gastrointestinal symptoms were diarrhea, anorexia, and nausea, in 24.2%, 17.9%, and 17.9%, respectively. A cohort study by Redd et al, in 9 hospitals in Massachusetts, United States, evaluated the presence of gastrointestinal symptoms in 318 adult patients (174 men) with COVID-19 at hospital admission and mean age 63.4 ± 16.6 years. A total of 195 (61.3%) patients presented with gastrointestinal symptoms upon admission, among which were anorexia (110; 34.8%), diarrhea (107; 33.7%), nausea (84; 26.4%), and vomiting (49; 15.4%).^[2]^ In a cohort study by Cholankeril et al, in an organization in the United States, gastrointestinal symptoms were reported in 31.9% (97) of patients with COVID-19, and the most common symptoms were loss of appetite (22; 25.3%), nausea/vomiting (12; 10.3%), and diarrhea (12; 10.3%).^[3]^

Moxibustion improves general health and treats chronic diseases such as arthritis and digestive system disorders by using the thermal stimulation produced by the burning of herbal preparations containing dried Artemisia argyi leaves or Artemisia argyi leaves on acupoints. Moxibustion is divided into direct moxibustion and indirect moxibustion.^[4]^ Among them, direct moxibustion involves placing the ignited moxa cone directly on the acupoint skin to ignite, which will cause pain and even scarring. Indirect moxibustion involves moxibustion of the ignited moxa cone at a certain distance from the skin, moxibustion of the cake made of salt, garlic and traditional Chinese medicine, or hanging the moxa cone on the needle and igniting it for moxibustion.^[5]^

In recent years, there have been an increasing number of reports about moxibustion treatment of anorexia in patients with COVID-19 (CO19-A), but there is no systematic review on moxibustion treatment of CO19-A. Therefore, we decided to fill the gap in the literature to provide experts and patients with up-to-date evidence that can be used to rigorously evaluate the effectiveness of this therapy and to guide clinical practice. We conducted this systematic review and meta-analysis to summarize the current evidence of the effects and safety of moxibustion therapy for the treatment of CO19-A.

## 2 Methods

### 2.1 Objectives and registration

This systematic review will aim to evaluate the effect and safety of moxibustion therapy for CO19-A. Our protocol has been registered on the International Platform of Registered Systematic Review and Meta-Analysis Protocols (PROSPERO). The registration number was CRD42022302499. All steps of this systematic review will be performed according to the Cochrane Handbook (5.2.0).

### 2.2 Ethics and dissemination plans

Given that there will be no patients recruited and no data gathered from patients, ethical approval is not necessary for our research. We will publish the results of this network meta-analysis in the form of journal papers or conference papers.

### 2.3 Eligibility criteria

PICOS principles will be consulted to establish the inclusion and exclusion criteria of this systematic review.

#### 2.3.1 Types of participants

Conditional studies: The patient was diagnosed as COVID-19, accompanied by anorexia, without any age, gender or limitation.

#### 2.3.2 Types of interventions and comparators

The series of moxibustion therapies involves many techniques, such as moxa stick moxibustion, moxa cone moxibustion, direct moxibustion, and indirect moxibustion. Moreover, many distinctive complex moxibustion manipulations are organically combined, such as partitioned moxibustion, moxa-moxibustion, and warm moxibustion.^[1]^ Studies that combine moxibustion with other therapies, such as acupuncture, massage, drugs, and physical interventions, will be included if they can prove that moxibustion is effective.

#### 2.3.3 Types of outcomes

The primary outcomes included the effective rate of clinical symptoms, body weight, and food intake on the gastrointestinal symptom rating scale (GSRS total score). The secondary outcomes will assess nausea and the incidence of adverse events.

#### 2.3.4 Types of studies

The selected articles should be randomized controlled trials comparing moxibustion and control groups to evaluate the efficacy of moxibustion on CO19-A. We will include an assessment of moxibustion compared with control interventions, including inactive controls (such as placebo, no treatment) and active controls (such as drugs and acupuncture). Conference literature and papers, reviews, case series, case reports, experience summaries and animal research will be excluded.

### 2.4 Data sources and retrieval strategy

We will search foreign and Chinese databases, including PubMed, EMBASE, MEDLINE, CENTRAL, CNKI, WanFang Data, CBM, and VIP from the inception of the coverage of these databases to December 2021.

Data, CBM, and VIP from the inception of the coverage of these databases to December 2020. The databases will be retrieved by combining the subject words with random words. Taking PubMed as an example, the retrieval strategy is shown in Table 1.

**Table 1:** PubMed search strategy.

Number	Search items
#1	“COVID-19” [Title/Abstract] OR“COVID 19” [Title/Abstract] OR“SARS-CoV-2 Infection” [Title/Abstract] OR “Infection, SARS-CoV-2” [Title/Abstract] OR“SARS CoV 2 Infection” [Title/Abstract] OR“SARS-CoV-2 Infections” [Title/Abstract] OR“2019 Novel Coronavirus Disease” [Title/Abstract] OR “2019 Novel Coronavirus Infection” [Title/Abstract] OR“2019-nCoV Disease” [Title/Abstract] OR“2019 nCoV Disease” [Title/Abstract] OR“2019-nCoV Diseases” [Title/Abstract] OR “Disease, 2019-nCoV” [Title/Abstract] OR “COVID-19 Virus Infection” [Title/Abstract] “COVID-19 Pandemics” [Title/Abstract] OR “COVID 19 Pandemic” [Title/Abstract] OR “2019-nCoV Infections” [Title/Abstract] OR “COVID-19 Virus Diseases” [Title/Abstract] OR “SARS Coronavirus 2 Infection” [Title/Abstract] OR “Disease 2019, Coronavirus” [Title/Abstract]
#2	“Anorexia” [Title/Abstract] OR“Anorexias” [Title/Abstract] OR “Na difference” [Title/Abstract] OR “dystrophy” [Title/Abstract] OR “mortification” [Title/Abstract] OR “food refusal”[Title/Abstract] OR “mental derangement”[Title/Abstract]OR “nausea” [Title/Abstract] OR “vomiting” [Title/Abstract]
#3	“Moxibustion” [title/abstract] or “Moxabustion” [title/abstract] or“moxa stick moxibustion”[title/abstract] or “moxa cone moxibustion”[title/abstract] or “direct moxibustion” [title/abstract]. or “indirect moxibustion” [title/abstract] or “partitioned moxibustion” [title/abstract] or“moxa-moxibustion” [title/abstract] or “warm moxibustion” [title/abstract]
#4	“andomized controlled trial” [Title/Abstract] OR“ randomized” [Title/Abstract] OR“ placebo”[Title/Abstract].
#5	#1 and#2 and #3 and #4

The search terms will be adapted appropriately to conform to the different syntax rules of the different databases.

### 2.5 Study selection and data extraction

**Figure 1:**
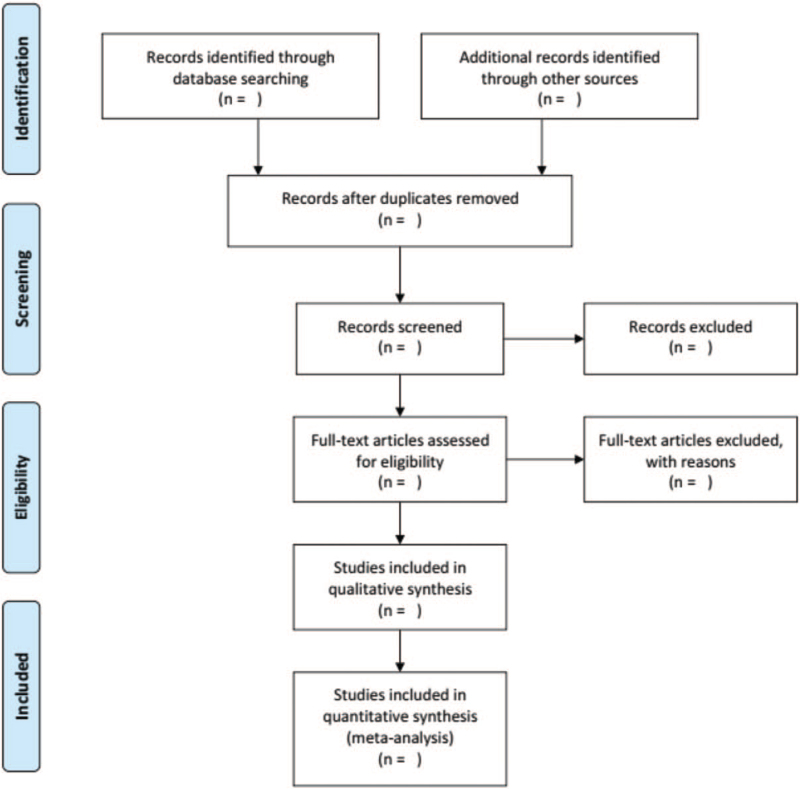
PRISMA flow chart.

EndNote X9 will be used to manage the retrieved studies. As shown in Figure 1, the study selection will be divided into 2 steps and completed by 2 researchers (YS and QY). Preliminary screening: Duplicate and irrelevant studies will be deleted while screening the titles and abstracts. Rescreening: We read through the full texts and select studies according to the inclusion and exclusion criteria.

According to the Cochrane Handbook for Systematic Reviews of Interventions, the 2 researchers (XXW and YH) will extract data, including the author, publication time, participant number, age, race, lesion location, intervention measures, course of treatment and outcome indicators, and they will enter these data in the data extraction table to compare results.

### 2.6 Risk of bias assessment

Two researchers (LD and YH) will assess the quality of the included clinical randomized controlled trials independently by utilizing the Cochrane Risk of Bias assessment tool. As specified by the Cochrane Handbook (5.2.0), the following sources of bias will be considered: random sequence generation, allocation concealment, participant blinding, outcome assessor blinding, incomplete outcome data, selective reporting, and other sources of bias. Each domain will be rated as having a high, low, or unclear risk of bias as appropriate.^[6]^ The 2 reviewers will resolve any disagreements through discussion, and a third reviewer (XW) will be consulted if no consensus is reached.

### 2.7 Statistical analysis

#### 2.7.1 Traditional meta-analysis

Direct comparisons of moxibustion efficacy will be performed using Review Manager 5.3. The outcomes will be mainly represented by the mean difference or odds ratio with 95% confidence intervals, and a *P* value <.05 will be considered significant. The Cochrane Q test and *I*^2^ statistics will be used to assess heterogeneity. When *P* < .1 or *I*^2^ > 50%, which indicates statistical heterogeneity, a random effects model will be used to calculate the outcomes; otherwise, a fixed effects model will be considered.

#### 2.7.2 Network meta-analysis

A network evidence diagram will be drawn to visually represent the comparisons between the studies. The size of the nodes represents the number of participants, and the thickness of the edges represents the number of comparisons. Stata 14.2 and WinBUGS 1.4.3 Software will be used to carry out Bayesian network meta-analysis. Bayesian inference will be carried out using the Markov chain Monte Carlo method, the posterior probability will be inferred from the prior probability, and estimation and inference will be assumed when Markov chain Monte Carlo reaches a stable convergence state. As a result, the rank of the moxibustion effect will be presented by the surface under the cumulative ranking curve.

Inconsistencies between direct and indirect comparisons will be evaluated using the node splitting method.^[7]^ The choices between fixed effects and random effect models and between consistent and inconsistent models will be made by comparing the deviance information criteria for each model.^[8,9]^

#### 2.7.3 Subgroup and sensitivity analysis

If the heterogeneity is high, we will also perform subgroup analysis to calculate the combined statistics.^[10]^ The following subgroup analyses will be considered: gender, age, intervention time, intervention cycle, and course of the disease.

When sufficient data are available, sensitivity analysis will be performed to test the robustness of the primary outcomes, which includes assessing the quality of the methods, the quality of the studies, and the impact of sample size and missing data.

#### 2.7.4 Publication biases

If 10 or more studies are included, we will use funnel plots to assess the level of publication bias. Asymmetry in the funnel plot will suggest the possibility of small study effects, and the results of the analysis will be interpreted cautiously.

### 2.8 Quality of evidence

The Grading of Recommendations Assessment, Development and Evaluation (GRADE) system will be used to assess the overall quality of the evidence derived from the included studies.^[11]^ In addition, the results will be divided into high, moderate, low and very low quality.

## 3 Discussion

Moxibustion, a very ancient modality of treating diseases, has been used throughout the history of human civilization and plays an important role in disease resistance. Moxibustion has been widely used for various conditions, including cancer, ulcerative colitis, stroke rehabilitation, constipation, hypertension, pain conditions and breech presentation. Although moxibustion is frequently used for CO19-A in practice, there has been no systematic study to inform current evidence on the effectiveness of moxibustion treatment for CO19-A. We hope that the results of this study may provide evidence regarding the moxibustion treatment of CO19-A.

## Author contributions

**Conceptualization:** Xingxin Wang, Jiguo Yang.

**Data curation:** Yi Hou, Lin Ding, Xiaojun Zheng, Yawen Sheng, Qiaoru Yu, Xiaoyun Bi.

**Formal analysis:** Xingxin Wang, Yi Hou.

**Methodology:** Jiguo Yang.

**Software:** Lin Ding, Xiaojun Zheng.

**Supervision:** Jiguo Yang.

**Writing – original draft:** Xingxin Wang.

**Writing – review & editing:** Jiguo Yang.
